# Analysis and Basics of Improving the Process of Cutting Electrical Sheet Bundles with a High-Pressure Abrasive Water Jet

**DOI:** 10.3390/ma17071666

**Published:** 2024-04-04

**Authors:** Monika Edyta Szada-Borzyszkowska, Wojciech Kacalak, Łukasz Bohdal, Wiesław Szada-Borzyszkowski

**Affiliations:** 1 Department of Mechanical Engineering, Faculty of Mechanical Engineering and Energy, Koszalin University of Technology, Racławicka 15-17 Street, 75-620 Koszalin, Poland; wojciech.kacalak@tu.koszalin.pl (W.K.); lukasz.bohdal@tu.koszalin.pl (Ł.B.); 2 Branch of the KUT in Szczecinek, Koszalin University of Technology, 78-400 Szczecinek, Poland; wieslaw.szada-borzyszkowski@tu.koszalin.pl

**Keywords:** electrical steel, high-pressure abrasive water jet cutting, cut surface, deformation zone, bundle cutting, magnetic properties

## Abstract

Electrical steels are widely used in the electrical industry in the construction of many devices, e.g., power transformer cores and distribution transformers. An important parameter of electrical components that determines the efficiency of devices is energy loss during remagnetization. These losses are influenced, among other factors, by steel cutting processes. The common techniques for cutting electrical materials on industrial lines are mechanical cutting and laser cutting. High-pressure abrasive water jet (AWJ) cutting, unlike the technologies mentioned above, can ensure higher quality of the cut edge and limit the negative impact of the cutting process on the magnetic properties of sheet metal. However, the correct control of the process and the conditions of its implementation comprise a complex issue and require extensive scientific research. This work presents a new approach to cutting electric sheets, involving bundle cutting, which significantly increases the processing efficiency and the dimensional and shape accuracy of the cut details. The tests were carried out for bundles composed of a maximum of 30 sheets, ready to be joined in a stator and rotor in a motor. The influence of processing conditions on the quality of the cut edges of sheet metal, the width of the deformation zone, and the burr height were analyzed. The detailed analysis of the quality of the cut edges of electrical bundled sheets creates new possibilities for controlling the AWJ cutting process in order to obtain a product with the desired functional and operational properties.

## 1. Introduction

Electrical sheet, also known as silicon or transformer steel, is a soft ferromagnetic material typically composed of low-carbon steel and commonly manufactured in cold-rolled strips. The laminates, which are crucial components for transformer cores or parts of stators and rotors in electric motors [1], are shaped using various cutting technologies such as blanking, guillotine cutting, electrical discharge machining (EDM), and laser cutting. Blanking and laser cutting processes are preferred due to their high efficiency and lower costs [2,3]. In the blanking process, material shaping occurs through plastic deformation using two cutting elements: a punch and a die. During the process, a zone of plastic deformation and material strengthening is generated, which, when excessively wide, deteriorates the magnetic properties by reducing permeability and increasing losses [4].

In [5], a comparison was made between the effects of abrasive waterjet (AWJ), wire electrical discharge machining (EDM), pulsed laser (PL), continuous wave (CW), and laser methods on single sheet metal. The AWJ treatment produced the most desirable magnetization curves, characterized by the highest saturation at the lowest applied field strength, resulting in the lowest core losses. In contrast, EDM and PL machining yielded magnetic properties with higher losses and lower saturation magnetization compared to those of AWJ machining. The CW-treated sample exhibited the least desirable magnetization curves, with significantly lower saturation and very high core losses. In the blanking process, the cutting edges of the tools gradually wear out [6]. The challenge lies in determining the critical degree of wear necessary to maintain defect-free cutting edges on electrical steel, preventing the formation of burrs that could potentially create interlayer short-circuit points in the cores of electric machine motors. Efforts are underway to explore methods of cutting electrical sheets and developing tools that mitigate defects in cut edges and preserve the magnetic properties of the sheets [7]. Studies [8,9] investigated the magnetic properties of electrical sheets following laser cutting, EDM, and blanking processes, considering the material’s condition after prior processing. An approximately 0.3 mm-wide zone of plastic deformation was observed at the edge of the cut material, a phenomenon absent in laser cutting. The authors demonstrated that cutting processes adversely affect the magnetic permeability of the sheets.

In laser cutting, induced thermal stresses are directly associated with increased energy losses and decreased magnetic permeability of electrical sheets [10]. The authors of [3] examined the impact of annealing at different temperatures on the microstructure of non-oriented sheets and their hysteresis loop characteristics following this process. It was discovered that these parameters are significantly affected by both the annealing temperature and duration. Furthermore, the effects of electrical discharge machining and guillotine cutting on selected magnetic properties of these materials were compared. In [11], the methods of laser and mechanical cutting for generator sheets were characterized. Sheets with silicon content ranging from 0.13% to 3% underwent testing. The study found that the impact of cutting technology on product quality was more pronounced following laser cutting compared to that of blanking. The authors attributed this difference to the concentration of thermal stresses near the cutting edge. The test results indicated that sheets with higher silicon content and larger grain are more susceptible to the adverse effects of the laser cutting process. These conclusions were corroborated by the authors of [5,12], who noted that this finding also extends to blanking processes.

One of the unconventional shaping methods currently utilized is abrasive machining with an abrasive water jet. It has the potential to replace traditional techniques for processing electrical sheets [13,14,15] and can be applied to a wide range of modern and conventional materials [16]. Knowledge about the processes of abrasive water jet cutting of magnetic materials is limited, and there is a scarcity of publications on this topic. In [10], the analysis focused on the influence of selected cutting technologies on the magnetic properties of non-oriented electrical steels, such as M800-65A, M800-50A, M400-65A, M400-50A, M300-35A, and NO20, commonly utilized in the production of electric machine cores. The authors employed mechanical, laser, water jet, and electrical discharge machining technologies. Through the creation of samples with varying widths, they investigated the influence of material degradation on the magnetic properties of the cut samples. Based on the research results, it can be concluded that water jet cutting and electrical discharge technologies facilitate the magnetization process of materials compared to the other analyzed technologies. This is attributed to the smaller values of stresses, strains, and thermal interactions in the material during these processes. However, the magnetization process, in addition to factors like grain size, is also influenced by the magnetic impurities present in the steel, which can immobilize domain walls [17,18]. Hence, achieving saturation state magnetization demands a stronger magnetizing field intensity. In [19], experimentation with the abrasive water jet cutting process on non-oriented FeSi sheet revealed a 1.5 mm wide deformed zone post-process, potentially exerting adverse effects on the material’s magnetic characteristics, including its magnetization curve and energy loss.

Abrasive water jet (AWJ) cutting stands out as a notably superior technology for magnetic materials compared to mechanical cutting methods. Its advantages include the elimination of the need to monitor cutting tool wear, a common requirement in blanking processes, as emphasized in [20]. The challenges related to cutting magnetic materials involve ensuring suitable process conditions to obtain a product of adequate technological quality [10,21]. When cutting thin laminates, increasing the erosion potential of the cutting jet by raising the working pressure of water, combined with an improper distance of the jet outlet from the processed material and an inappropriate feed speed, may lead to defects in the cut edge. These defects include heterogeneity of the cutting surface, excessive roughness, transverse cracks, burrs, damage to the electrical insulating coating, and deviations in the shape of the cut edge [22].

The AWJ cutting process, particularly at high cutting speeds, induces plastic deformations concentrated near the cutting edge, resulting in a smaller deformation zone compared to that of mechanical cutting processes. This reduction mitigates adverse changes in coercive intensity and remanence induction. Studies have shown that optimal processing parameters in mechanical cutting can even lead to increased remanence induction compared to that of the highest AWJ cutting speeds [23]. The cost of abrasive materials does not pose a significant limitation to the development of abrasive water jet (AWJ) technology across various applications [24]. Furthermore, recycling these materials reduces costs, rendering the technology more economically viable, efficient, and environmentally friendly, thereby expanding its potential applications [25]. The stator and rotor components in a motor consist of hundreds of joined and laminated electrical steel sheets [26]. Joining these laminated electrical steels for motor applications poses a highly complex challenge. The purpose of joining laminated electrical steels is to ensure the mechanical strength of the laminates [27]. However, the joining process can lead to a deterioration of magnetic properties due to damage to the insulating coating [28], modification of the microstructure [29], and the introduction of residual stresses [30]. Bundle cutting with a high-pressure abrasive water jet offers the opportunity to obtain a finished stack of laminates, ready to be assembled in the stator and rotor of a motor, with exceptional dimensional accuracy and devoid of shape deviations, burrs, and other defects typically encountered in blanking or cutting individual sheets. This approach can effectively mitigate the deterioration of magnetic properties at the initial stage of production and streamline the subsequent joining process. The aim of this study is to investigate the influence of cutting conditions on the geometric structure of the surface of electrical sheet bundles after cutting with a high-pressure water-abrasive jet.

## 2. Methodology and Experimental Studies

### 2.1. Features of the Waterjet Cutting Process

A high-pressure water jet of 366.8 MPa flows from a water nozzle with a diameter of 0.3556 mm at a speed of 856 m/s. Subsequently, the jet enters the mixing chamber where it combines with abrasive particles generated in a mixing nozzle with a diameter of 0.762 mm. The abrasive particles acquire significant kinetic energy and travel with the jet at a speed of 823 m/s. The theoretical description of this phenomenon, particularly the development of an appropriate model for the entry of abrasive grains into the water jet, is highly complex. A number of simplifying assumptions are employed to describe the movement of grains in the stream. These include assuming that the abrasive grains are spherical in shape and that there is no direct interaction between them. Additionally, it is assumed that in the absence of axial and radial flow, the movement of grains in the stream is steady and characterized by an initial speed equal to zero. The power during linear cutting is 28 kW.

Several stations, each equipped with the necessary instrumentation and software, were utilized to execute the full experimental research plan. Selection of parameters was crucial for ensuring the accuracy of measurements in each process.

### 2.2. Test Stand for Cutting Electrical Sheet Bundles with an AWJ Jet

Recent scientific achievements have laid the foundation for the dynamic development of modern technologies. The issue of cutting materials to achieve satisfactory quality while reducing production process time is essential for economic savings. In this regard, the technology of cutting with a high-pressure abrasive water jet (AWJ) deserves special attention. The unique properties of the AWJ jet, along with previous exploratory research [23], have provided the groundwork for further investigation into its use in cutting electrical sheets.

**Figure 1:**
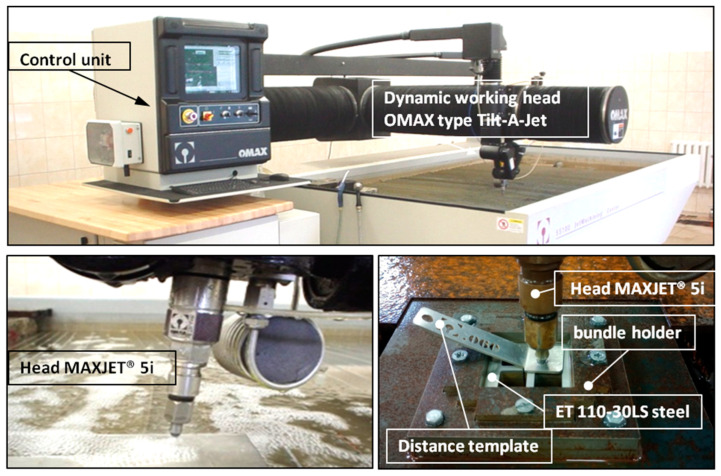
The JetMachining Center 55100 hydrojet machining center equipped with a Tilt-A-Jet head.

The analysis of previous exploratory research has confirmed that cutting electrical sheet metal using a high-pressure abrasive water jet (AWJ) is an effective method to achieve satisfactory surface quality of the cut material. The cutting of electrical sheet bundles was conducted using the OMAX Jet Machining Center 55100/4055V hydrojet machining center from the American company OMAX Corporation, Kent, WA, USA (refer to Figure 1). Material processing was performed using a cutting head equipped with a 5-axis Tilt-A-Jet system, enabling high dimensional and shape accuracy of ±0.05 mm. The machine is equipped with a high-power, high-pressure plunger pump (30 kW), capable of producing a water jet with a maximum pressure of p_max_ = 385 MPa and a volumetric efficiency of Q_max_ = 0.065 dm^3^/s.

The dynamic three-dimensional shaping mechanism of the Tilt-A-Jet is equipped with a water nozzle with a diameter of 0.3556 mm and an OMAX MaxJet 5 concentrating nozzle with a diameter of 0.762 mm. The electrical sheet was pre-cut into 70 × 70 mm formats. These prepared sheet metal bundles, consisting of 10, 20, and 30 pieces, were securely held in place using a special mounting device to prevent movement during cutting with the AWJ jet. The abrasive material used in the jet was garnet with a granulation of 80 mesh, with the basic parameters provided in Table 1.

The distance of the nozzle from the workpiece during cutting was consistently set to 0.06 inches. From each prepared bundle, four samples measuring 14 × 14 mm were cut. The cutting process was conducted at a working pressure of 366.8 MPa, with an abrasive flow rate of 0.265 kg/min.

### 2.3. A Station for Assessing the Geometric Structure of Surfaces and Shape Deviations

Confocal laser scanning microscopy technology was employed to assess the surface of the cut edges. In the experimental studies, advanced measurement technology was utilized, employing the LEXT OLS4000 laser confocal microscope from Olympus (manufactured by Olympus, Tokyo, Japan). The basic parameters of this microscope are presented in Table 2.

**Figure 2:**
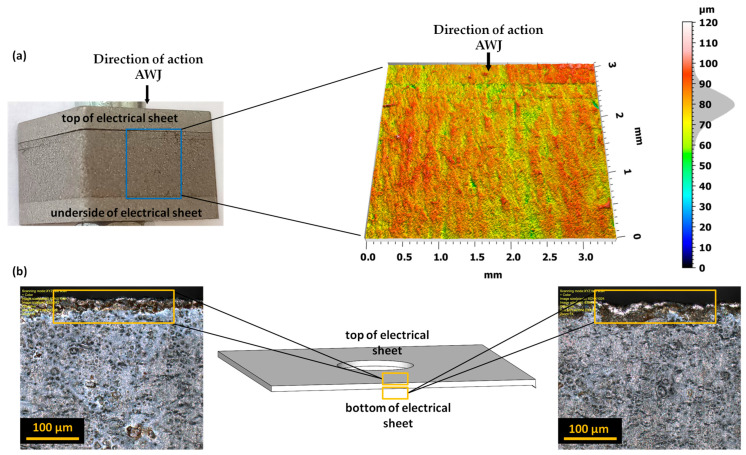
The measurement areas include (**a**) a view of the bundle of electrical sheets with stainless steel sheets on both sides used for compression, displayed on the left side, and a view of the scanned surface of the sheet bundle on the right side; (**b**) a view of the upper and lower edges of a single electrical sheet.

Precise scanning in the x-y axes made it possible to obtain a spatial representation of the examined edges of bundles and electrical sheets. The advanced measurement system allows for measuring the surface of elements with a large surface inclination angle of up to 85° and a low reflection coefficient. A set of 5 motorized microscope objectives in a special revolver holder allows for performing measurements at magnifications of 5×, 10×, 20×, 50×, and 100×. The additional possible optical magnification (zoom) allows for magnifications ranging from 1× to 8×. The total magnification that could be obtained was up to 17,280×. Placing the samples on a motorized measuring table allowed for displacements in the *x*–*y* axes (range: 120 mm) and *z*-axis (range: 10 mm) in the field of observation from 16 × 16 µm to 2560 × 2560 µm. The BF/DIC/Laser/DIC confocal laser observation method was used to observe the surface. Processing and analysis of recorded images were carried out using LEXT 5.0 software, provided by the device manufacturer. To assess the edges of bundles consisting of 10, 20, and 30 sheets of electric steel ET 110-30LS after cutting with a high-pressure abrasive water jet, microscope settings were used with a 20× objective and a ×433 magnification. The measurement was made from the front of the sample in the bundle; then, each sample was unfolded, and the measurement was made on the upper and lower edges of the sample. Figure 2 displays a cut sample of a bundle comprising 10 sheets positioned between upper and lower 2 mm thick sheets. An enlarged image depicting the geometric structure of the surface, along with an edge height map, is presented. Subsequently, the sheets within the bundle were separated to analyze the deformation zone and measure burrs. Burrs and the deformation zone of electrical sheets play a crucial role in the construction of transformer cores, as their shape and height can influence the formation of potential interlayer short-circuit points. The analysis of the obtained results was carried out using the TalyMap Platinum program (version 7.4).

The Phenom ProX scanning electron microscope (Thermo Fisher Inc., Waltham, MA, USA) was used to scan the surface with an SEM electron beam. The basic parameters of the Phenom ProX are presented in Table 3.

Taking high-quality electron photos in a short time is achieved through automatic focusing and astigmatism correction. The color navigation camera enables perfect correlation between electronic and optical images.

### 2.4. Material

Cold-rolled grain-oriented electrical sheet made of ET 110-30LS steel with a thickness of 0.3 mm (0.012 inch) and a minimum induction of B_800_ = 1.87 T was used for the tests. The sheet supplier provided the appropriate certificates. These sheets are covered on both sides with a thin gray electrical insulating coating with a thickness of 1.5–3.0 μm (marked according to ASTM A976-13:2018 [31]), which is resistant to annealing at temperatures up to 840 °C in a non-oxidizing atmosphere. This coating has good adhesion to the substrate, ensuring very good insulation resistance (>15 Ω/cm^2^, measured in accordance with the IEC 60404-11:2012 standard [32]). Additionally, the sheet metal underwent laboratory tests to measure its mechanical properties, carried out on a Zwick/Roell Z400 testing machine. The results are summarized in Table 4.

These sheets are primarily utilized in the cores of power and distribution transformers. Moreover, they find application in the production of various components such as audio transformers, voltage transformers, current relays, magnetic screens, wound cores, and medium to large high-efficiency generators and reactors. The utilization of precisely cut electrical sheet metal is crucial for achieving optimal magnetic parameters, as the quality of the cut surfaces and edges significantly impacts performance.

### 2.5. AWJ Process Parameters

The analysis of previous exploratory research confirmed that the optimal cutting parameters with the AWJ were obtained at the lowest cutting speed [23]. These parameters are presented in Table 5.

The initial formatting of the electrical sheet into 70 × 70 mm formats was carried out using identical parameters to those of the actual tests. The prepared sheets were then placed in a clamping device to form bundles of 10, 20, and 30 sheets each. A 2 mm thick AISI 304 stainless steel sheet was inserted on both sides of every bundle. The use of an additional sheet of metal allowed for even pressure of the sheets in the bundle using screws. After compressing the bundle, the bundle holder was placed in the working space of the OMAX Jet Machining Center 55100/4055V hydrojet machining center and secured against movement during cutting. Four samples of 14 × 14 mm were cut from each bundle prepared in this way. Before cutting out each sample, a hole was made in its central place and tightened with a screw. This allowed it to be held together after the cutting process and in subsequent tests. Table 6 presents a summary of the speeds of the waterjet cutting process. The different thicknesses of each bundle resulted in a change in the cutting speed.

After drying, the samples underwent subsequent microscopic analysis using the OLYMPUS LEXT OLS4000 (Tokyo, Japan) confocal laser microscope with a 20× objective and a 433× magnification, followed by analysis using the Phenom ProX scanning electron microscope (Thermo Fisher Inc., Waltham, MA, USA).

## 3. Experimental Results

Bundle cutting becomes unprofitable when performed using mechanical blanking [33,34]. In [34], the author conducted a multi-layer blanking process on electrical steel laminates consisting of six sheets with a total stack thickness of 2.1 mm. They highlighted issues related to selecting appropriate cutting clearance, punches, and dies capable of withstanding heavy loads, as well as concerns regarding the low quality of the cut edges of the resulting layers and the rapid wear of cutting tool edges. Given the need for precise dimensions and tolerances of laminates in stator core production, multi-layer blanking demands highly accurate manufacturing of punches and dies, along with the design of appropriate process kinematics. The results obtained indicate that even slight wear of cutting tools can result in the formation of burrs, deformation zones, and the welding of sheet metal stacks [34,35]. Cutting sheet metal bundles using the AWJ method with appropriate pressure in the stack ensures that plastic deformations inside the material are limited and that conditions conducive to the formation of burrs and built-up edges are avoided [36]. As a result, the quality of the cut edges of stacked sheets is much higher compared to that of multi-layer blanking [37].

Table 7, Table 8 and Table 9 present the basic parameters describing the geometric structure of the surface for bundles consisting of 10 sheets (Table 7), 20 sheets (Table 8), and 30 sheets (Table 9) of electrical sheets.

The analysis of individual parameters of the geometric structure of the surface indicates that reducing the height of the rises (Sp) can be achieved by increasing the number of sheets in the bundle to 30. The highest values of the Sp parameter were observed for sheets in the range of 10 to 30, counted from the top of the bundle surface. Notably, higher Sp values were found for sheets with larger gaps between them, especially gaps exceeding 100 µm. These protruding gaps typically stem from local deviations in the flatness of the sheets, underscoring the significance of the quality of prepared bundles in determining the quality of edges and surfaces after cutting. However, it is important to note that bundling helps reduce the occurrence of burrs and material outflows.

Furthermore, the analysis of cavities located below the Sv core profile—the lowest part of the surface—revealed an increase in cavities when the number of sheets in the bundle was increased to 30. Similar to the Sp parameter, the most favorable values of the Sv parameter were found for sheets in the range of 10 to 30. Interestingly, for sheets with larger gaps in the bundle, the Sv parameter exhibited a slight decrease.

**Figure 3:**
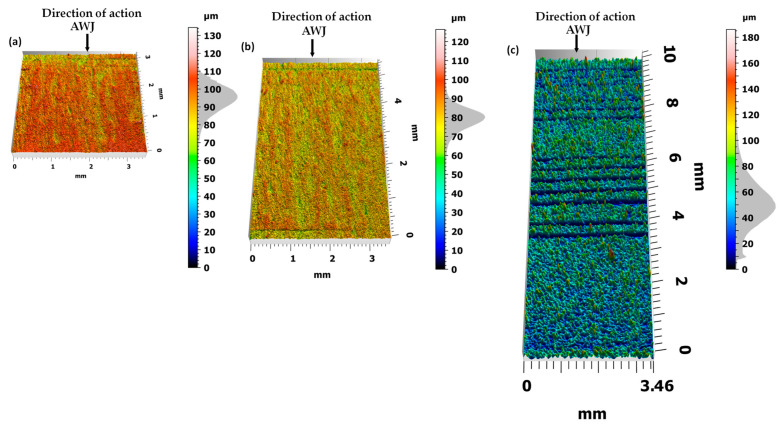
The view of the cut surface after cutting (**a**) 10 sheets, (**b**) 20 sheets, and (**c**) 30 sheets in a bundle.

After cutting with an AWJ (Figure 3), each surface of bundles containing 10, 20, and 30 sheets of electrical sheets exhibits specific microgeometric irregularities. In bundles of 10 sheets, the maximum unevenness reaches approximately 135 μm, with an average value of 95 μm and a standard deviation of ±15 μm. In bundles of 20 sheets, the largest irregularities are around 130 μm, with an average value of 80 μm and a standard deviation of ±15 μm. However, in bundles of 30 sheets, the largest irregularities measure approximately 185 μm, with an average value of 50 μm and a standard deviation of ±40 μm.

On the cut surface, especially in the area of the last sheets, one can observe structure distortion in the form of grooves and microgrooves. As the distance from the jet outlet from the cutting head increases, the kinetic energy of the jet decreases, making the curved path of the cutting beam visible.

This effect of the abrasive is caused by the characteristics of the jet when cutting materials at different feed speeds. The characteristic structure in the form of curved grooves, which can be observed on all analyzed bundles, also becomes visible as the jet feed increases.

A less wavy and irregular surface at lower feed speeds results from a larger number of abrasive grains shaping the machined surface. This leads to the removal of excessive flashes on the lower edges of the sheet. The quality of the cutting surface scale largely depends on the conditions of the AWJ cutting process. Each feature can therefore be eliminated or minimized by appropriately selecting cutting parameters. A preliminary analysis of these parameters was performed in previous experimental studies [23].

**Figure 4:**
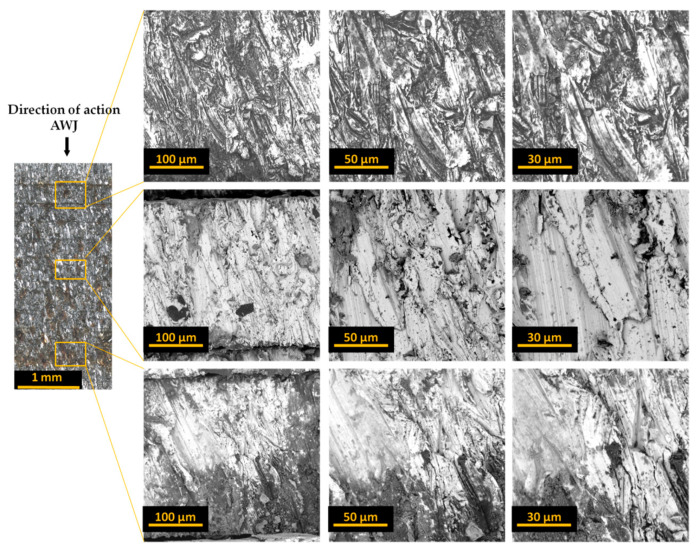
Images of the cut surface recorded for a bundle containing 10 sheets.

**Figure 5:**
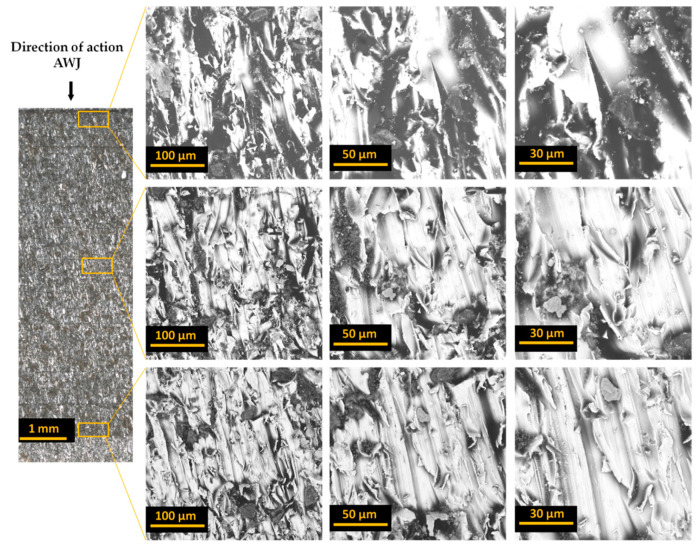
Images of the cut surface recorded for a bundle containing 20 sheets.

**Figure 6:**
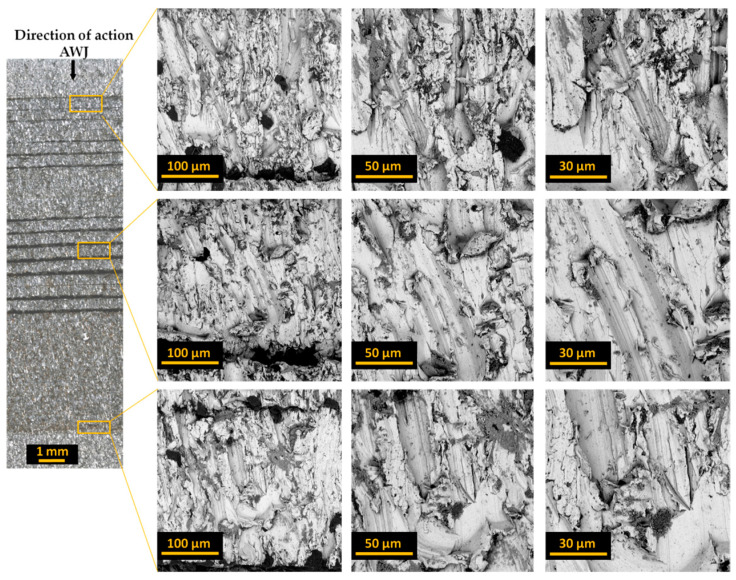
Images of the cut surface recorded for a bundle containing 30 sheets.

Figure 4, Figure 5 and Figure 6 present images of the surface after cutting with an AWJ obtained at various magnifications. These images were captured by scanning them with a focused SEM electron beam of the Phenom ProX microscope using a backscattered electron detector.

Images of entire bundles and selected sheets of electrical steel are presented for bundles of 10 (Figure 4), 20 (Figure 5), and 30 (Figure 6). The initial zone is the zone where the jet enters the processed material. In this zone, the processed bundle of electrical sheets comes into direct contact with the sheet pressing the bundle. In this zone, there is no effect of jet deflection on the processed surface. The next presented zone (middle zone) was the area where the beveling of the high-pressure abrasive water jet begins, observed already at half the thickness of the bundle. The last zone (end zone) includes the last sheets of the bundles, where there is a change in the direction of traces of the impact of the deflection of the high-pressure water jet.

Analysis of the obtained images of the entire bundles showed the presence of noticeable gaps between some sheets. In the analyzed samples, this is most often visible for sheets with initially occurring deformation and waviness of the sheet from which the forms are cut. These unevennesses cause microgaps to occur when packing the sheets. During the cutting process with an AWJ jet, especially in its first stage when piercing through the material, water and abrasive are reflected from subsequent sheets of the bundle. Every smallest gap between the sheets is a space where processed material and abrasive grains can accumulate. This may cause the gap to widen. Therefore, the flatness of the sheets and their adhesion to each other in the bundle are of great importance for the quality of processing.

The presented images of selected sheets from the sheet bundle also confirm the occurrence of jet deflection in the direction opposite to that of the moving cutting head. This outflow of the stream, with a certain delay in relation to the point of its entry into the material, results in patterns on the cut surface in the form of usually parallel curved grooves.

**Figure 7:**
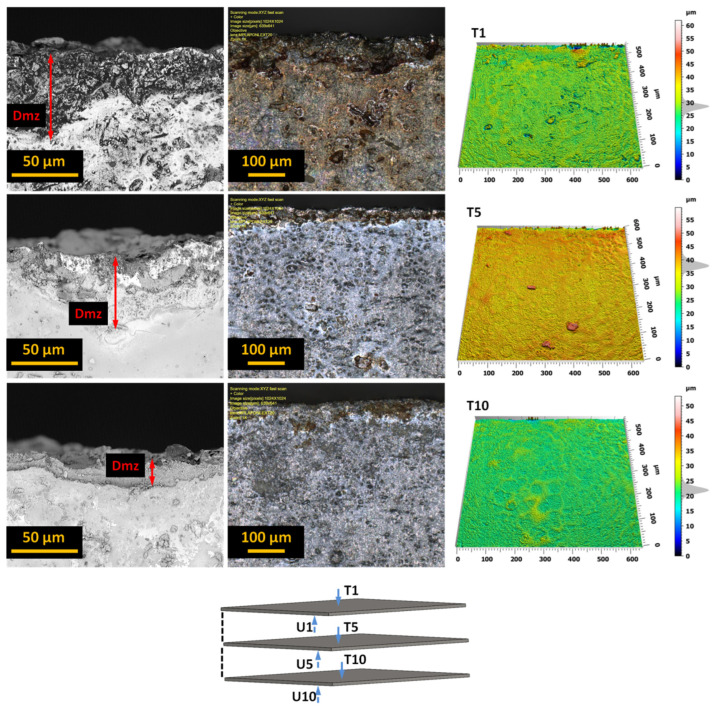
The upper surface of the edges of selected sheets (T1, T5, T10) from a bundle of 10 sheets with marked deformation zones.

In the next stage of the research, each bundle was disassembled and analyzed regarding the formation of burr height on the edges of the sheets. For example, Figure 7 shows different views of these edges for a selected sheet from a bundle of 10 sheets. It presents views of the upper surface of selected sheets (1, 5, 10) in a given bundle.

**Figure 8:**
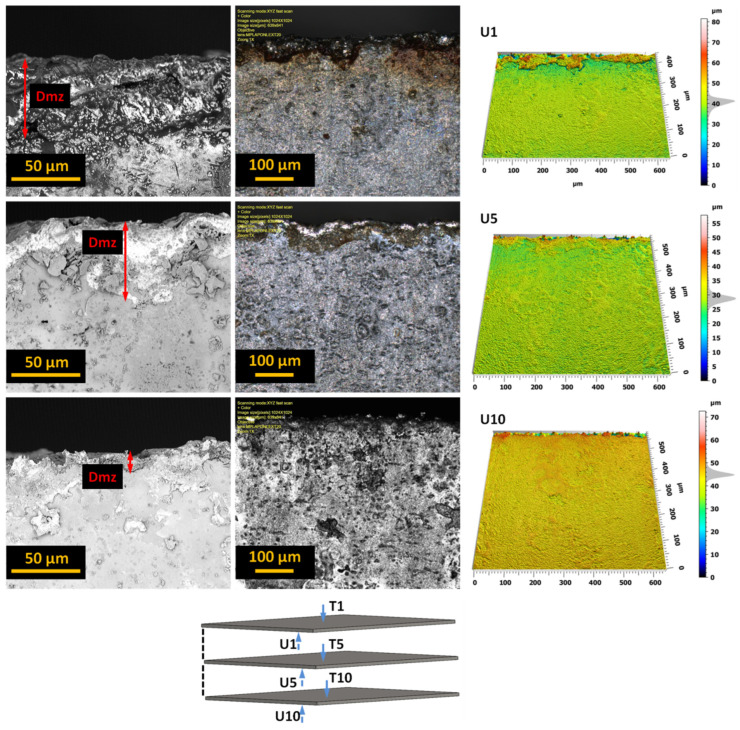
The lower surface of the edge of selected sheets (U1, U5, U10) from a bundle of 10 sheets with marked zones of deformation and occurring burrs.

The analysis of the obtained images revealed the presence of a deformation zone on the sheet surface. However, the size of this zone does not exceed 50 μm, and similar results were obtained for other tested sheets. More interesting effects regarding the burr height were observed after analyzing the edge opposite to the direction of entry of the AWJ jet, treating this side as the bottom of the sheet. Figure 8 presents examples of different views of these edges for a selected sheet from a bundle of 10 sheets.

The analysis of the obtained images allowed us to assess the size (height and width) and nature of the resulting burrs. We can also observe the width of the machining buildup formed in the jet exit zone. The analysis and influence of the burr width on the properties of electrical sheets are planned in subsequent studies. The heights of the burrs created when cutting the bundles with the AWJ are well below 25 μm.

**Figure 9:**
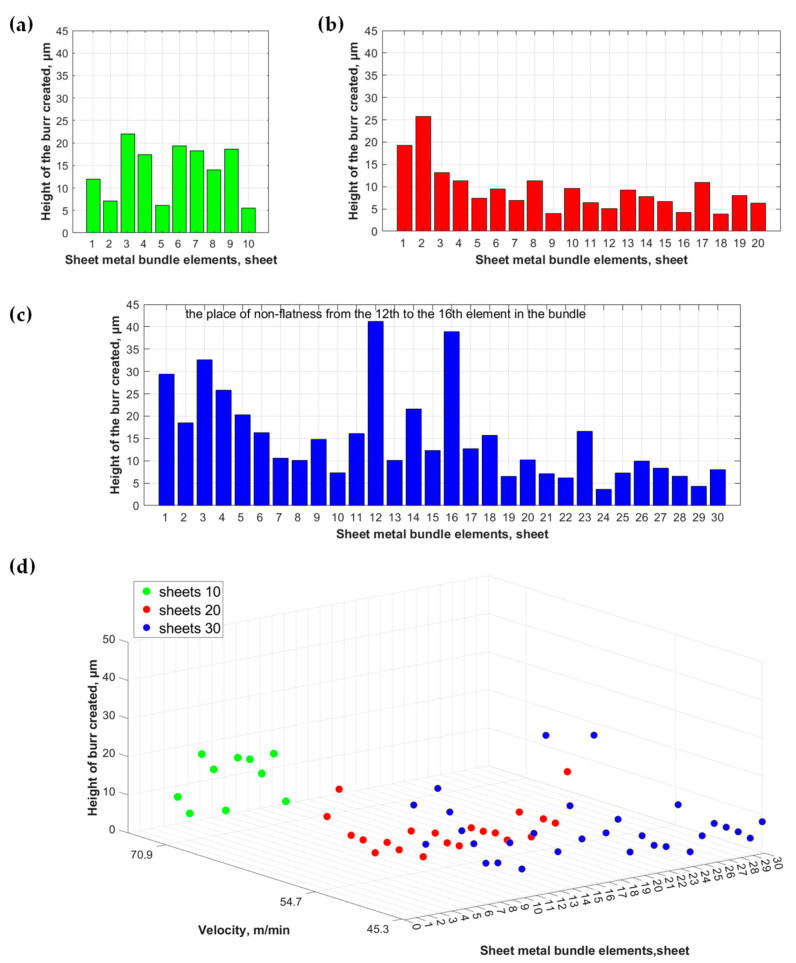
The burr height for individual sheets of the analyzed electrical sheet bundles: (**a**) for a bundle of 10 sheets, (**b**) for a bundle of 20 sheets, and (**c**) for a bundle of 30 sheets. Additionally, (**d**) provides a list of the burr heights for each sheet from bundles of 10, 20, and 30 sheets.

Figure 9 presents a summary of the burr height for subsequent electrical sheets in each of the analyzed bundles.

A comprehensive analysis of machining growths revealed that there are sheets for which, after the AWJ cutting process, the heights of the resulting burrs significantly exceed the values obtained for the remaining sheets of the bundle. Comparing images of the cut surfaces of the bundles with the height of the burrs on the edges of individual sheets showed that the largest burrs occur on the edges of the sheets between which there are the previously described gaps larger than 20 μm.

## 4. Conclusions

The cutting process using AWJ is complex, and the current knowledge about its course and the quality of the cutting edge obtained in the case of electrical sheets is limited. This technology requires precise definition of the process conditions and correct selection of parameters to obtain products of appropriate technological quality.

This work presents a new approach involving cutting out details consisting of bundles of ET 110-30LS electrical sheets ready to be integrated into a stator and rotor in a motor using a high-pressure abrasive water jet. This approach enables a significant increase in machining efficiency while maintaining very high dimensional and shape accuracy. The results of testing the quality of the cut edge depending on the number of sheets included in the bundle are presented. The following conclusions emerge from the research conducted:

- No defects were found in the cut edge of each prepared bundle, i.e., those consisting of 10, 20, and 30 sheets, which would impede the use of the respective laminate in electric motor core production;
- The AWJ process generates a localized deformation zone that could potentially impact the micromechanical, microstructural, and magnetic properties of the workpiece near the cutting edge. However, the width of this zone does not exceed 50 μm, which, based on the literature data and our own exploratory research, is significantly narrower compared to values observed in laser cutting and blanking processes;
- Only in two sheets isolated from a bundle of 30 sheets were burrs with a height of approximately 40 μm found, resulting from a microgap between the sheets. However, even in this case, the maximum burr height obtained as a result of AWJ cutting is similar to that obtained in the blanking process and does not result in waste. To achieve the highest quality in the multilayer cutting process, it is justified to optimize the bundle thickness, preceded by a statistical analysis of the sheet flatness.

The detailed analysis of the quality of cut edges in electrical bundled sheets opens up new possibilities for controlling the AWJ cutting process to achieve a product with the desired functional and operational properties.

## Figures and Tables

**Table 1:** Properties of the abrasive used in the AWJ jet cutting process.

Parameters	Values
Density, kg/m^3^	3500 to 4250
Bulk density, kg/m^3^	2200 to 2500
Hardness on the Mosh scale	7 to 7.5

**Table 2:** System specifications of the LEXT OLS4000.

Parameters	Values
Objective Lens Magnification	5× to 100×
Laser 3D image Magnifications	20× to 100×
Total Magnification capability	5× to 17.280×
Digital Magnification	1× to 8×
Color Imaging Mode	White LED Light
Laser Imaging Mode, nm	405
Minimum XY-Resolution, nm	120
Minimum Z-Resolution, nm	10
Field of view, µm	16 × 16 to 2560 × 2560

**Table 3:** Basic parameters of the Phenom ProX.

Parameters	Values
Light optical magnification	27× to 160×
Electron optical magnification range	160× to 350,000×
Resolution, nm	≤6 SED and ≤8 BSD
Digital zoom	max. 12×
Acceleration voltages, kV	5 ÷ 20
Sample size, mm	Up to 25 (optional 32)
Sample height, mm	Up to 35 (optional 100 mm)
Lifetime of thermionic source (CeB6), hour	to 1500

**Table 4:** Basic mechanical properties of ET 110-30LS steel (at *T* = 20 °C).

Density [kg/dm^3^]	R_m_ [MPa]	F_m_ [kN]	A_g_ [%]	R_p0,2_ [MPa]	Hardness [HV]	Hardness [HB]
7.8	337	0.96	10.93	314	165	157

**Table 5:** AWJ process parameters.

Parameters	Values
High pressure setting, MPa	366.8
Abrasive flow rate, kg/min	0.265
Orifice diameter, mm	0.3556
Mixing tube diameter, mm	0.762
Abrasive size, mesh	80
Standoff distance, mm	1.5

**Table 6:** Feed speed of the process of cutting electrical sheet bundles with an AWJ.

lp	Pressure p, MPa	Number of Sheets in the Bundle pcs	Traverse Speed m/min
1	366.8	10	70.9
2		20	54.7
3		30	45.3

**Table 7:** The SGP parameters of electrical sheets for a bundle of 10 sheets.

Nr Arkusza	Sp, [µm]	Sv, [µm]	Sz, [µm]	Sa, [µm]
1	50.21	78.4	128.7	4.52
2	60.7	97.3	158.0	6.73
3	21.3	119.5	140.8	4.83
4	5.58	159.9	165.5	4.85
5	14.82	150.0	164.8	5.22
6	59.4	75.1	134.6	5.90
7	36.8	117.1	154.0	6.52
8	59.4	75.1	134.6	5.96
9	53.7	82.9	136.6	6.16
10	48.9	102.6	151.5	6.40

**Table 8:** The SGP parameters of electrical sheets for a bundle of 20 sheets.

Nr Arkusza	Sp, [µm]	Sv, [µm]	Sz, [µm]	Sa, [µm]
1	73.8	136.7	210.5	5.83
2	106.2	119.6	225.7	6.14
3	91.3	123.1	214.4	5.23
4	103.4	98.3	201.7	5.85
5	90.1	107.4	197.6	5.60
6	69.36	147.3	216.7	4.73
7	55.4	140.5	196.0	4.46
8	73.0	128.8	201.6	4.66
9	72.6	106.6	179.3	4.78
10	79.9	105.4	185.4	5.33
11	89.3	99.9	189.4	4.7
12	64.3	129.7	194.0	5.15
13	56.8	114.4	171.2	5.03
14	61.8	71.9	133.8	5.64
15	57.3	122.0	179.3	4.31
16	56.2	137.5	193.7	4.43
17	60.3	132.7	193.0	4.56
18	64.7	127.6	192.4	4.66
19	138.6	73.7	212.3	4.96
20	140.5	8.8	222.4	6.17

**Table 9:** The SGP parameters of electrical sheets for a bundle of 30 sheets.

Nr Arkusza	Sp, [µm]	Sv, [µm]	Sz, [µm]	Sa, [µm]
1	67.8	81.5	149.8	5.37
2	61.5	72.0	133.6	4.51
3	84.6	88.1	172.8	5.20
4	96.8	141.8	238.1	5.62
5	85.9	97.8	183.8	4.54
6	71.8	119.2	191.0	5.82
7	59.3	102.2	161.5	6.57
8	56.9	101.1	158.1	6.39
9	71.4	117.6	189.1	6.40
10	48.8	82.3	131.2	5.34
11	54.7	120.1	174.8	5.26
12	57.8	150.0	207.1	4.08
13	86.4	106.0	192.5	6.00
14	97.1	141.0	238.1	5.51
15	68.2	106.8	175.1	5.61
16	55.3	104.4	159.7	6.45
17	41.5	168.5	210.0	5.12
18	49.7	142.2	191.9	5.86
19	75.3	110.2	186.6	5.36
20	71.3	117.8	189.1	6.38
21	48.6	82.5	131.2	5.37
22	53.3	136.5	189.8	4.90
23	30.6	158.8	189.4	4.77
24	28.5	146.7	175.3	4.61
25	26.2	186.9	213.2	5.04
26	21.8	215.1	237.0	4.90
27	23.8	164.4	188.2	5.60
28	23.6	153.5	177.1	5.04
29	20.7	159.2	179.9	4.93
30	16.8	220.4	237.2	4.43

## Data Availability

Data are contained within the article.
